# Characteristics, Treatments, and Survival of Uveal Melanoma: A Comparison between Chinese and American Cohorts

**DOI:** 10.3390/cancers14163960

**Published:** 2022-08-17

**Authors:** Jingting Luo, Chengkai Zhang, Yuhang Yang, Jingying Xiu, Hanqing Zhao, Chuqiao Liang, Zhaoxun Feng, Yuning Chen, Yueming Liu, Yang Li, Wenbin Wei

**Affiliations:** 1 Beijing Tongren Eye Center, Beijing Key Laboratory of Intraocular Tumor Diagnosis and Treatment, Beijing Ophthalmology & Visual Sciences Key Laboratory, Beijing 100730, China; 2 Medical Artificial Intelligence Research and Verification Key Laboratory of the Ministry of Industry and Information Technology, Beijing Tongren Hospital, Capital Medical University, Beijing 100730, China; 3 Department of Neurosurgery, Beijing Tiantan Hospital, Capital Medical University, Beijing 100730, China; 4 Department of Ophthalmology, University of Ottawa, 501 Smyth Rd, Ottawa, ON K1H 8M2, Canada

**Keywords:** uveal melanoma, Chinese, American, characteristics, treatments, survival

## Abstract

**Simple Summary:**

This retrospective comparative cohort study aimed to determine whether there were racial or national differences in UM, by comparing the demographic and clinical characteristics, such as tumor size, onset age, trend and proportion of treatment modalities, and overall survival. In the two cohorts, we found that Chinese patients have a younger onset age and a better survival rate. The survival advantage was likely secondary to younger onset age. In addition, a greater proportion of Chinese patients received brachytherapy as opposed to enucleation compared with American patients. This study was the first time comparing patients from different countries and races, which may help ophthalmologists better understand the clinical characteristics of the disease and suggests the importance of early diagnosis and treatment.

**Abstract:**

Uveal melanoma (UM) is the most common intraocular malignant carcinoma. This study aimed to compare the clinical features, treatment modalities, and prognosis of UM patients in China with those in America over a 15-year period. In the study, 4088 American patients with primary UM from the Surveillance, Epidemiology, and End Results (SEER) database and 1508 Chinese patients from Tongren-ophthalmology Research Association of Clinical Evaluation (TRACE) were included. Univariable and multivariable analyses were performed to determine prognostic factors and propensity score matching (PSM) and sensitivity analyses were applied to adjust for confounders and identify independent prognostic factors. Chinese patients were diagnosed at a younger age (mean ± SD, 47.3 ± 12.5 years vs. 59.7 ± 14.8 years) and tumors at diagnosis were larger (diameter: 12.0 ± 3.54 mm vs. 11.3 ± 8.27 mm; thickness: 7.13 ± 3.28 mm vs. 4.91 ± 3.01 mm). Chinese patients were more likely to undergo brachytherapy than American patients. Chinese patients had better overall survival than American patients while no significant differences exhibited after adjusting for age through PSM. In conclusion, compared with American patients, Chinese patients had younger onset age, larger tumors at diagnosis and better prognosis, mainly because of their younger age.

## 1. Introduction

Uveal melanoma (UM) is the most common primary intraocular malignant tumor in adults [1]. There is a known racial predilection, with higher incidence in Caucasians (5–6 per million) than Asians (0.4–0.6 per million) and Africans (0.3 per million) [2,3,4]. Previous studies have demonstrated variable clinical characteristics and outcomes of UM among different ethnicities [5,6,7]. In a retrospective study of 8100 American patients with UM, when compared to Caucasian Americans, Asian Americans had younger age at diagnosis, the tumor farther away from the fovea and optic disc and greater likelihood of rupture of Bruch’s membrane. In a literature review of Asian patients with UM, Manchegowda et al. showed that the tumor in Asians had larger base diameters than Caucasians with median-sized tumor being the most common [3].

Ocular treatment for UM consists of globe-preserving therapies (i.e., brachytherapy (BT), proton beam radiotherapy, transpupillary thermotherapy, surgical resection) or enucleation (EN). BT and EN are the most used modalities worldwide including in China [8]. Charged-particle radiotherapy and stereotactic radiotherapy, although effective, are less commonly used in China and other developing countries owing to their high cost [9]. Despite effective therapy for ocular tumors, outcomes for metastatic UM remain dismal due to a lack of effective treatment [10]. Treatment options may vary by region based on expertise and resources available. Consequently, survival disparity may be due to variations in tumor characteristics, treatment and social economic determinants.

Although previous studies have compared the characteristics and treatment differences of UM between racial groups in multicultural countries like America, there is a paucity of study focused on international comparison using large databases from two countries. Beijing Tongren Hospital was founded in 1886 and is one of the oldest and largest eye hospitals in China. We receive referrals of patients with UM from 32 provinces across China, which enabled us to accrue a large cohort of Asian UM patients [11]. Through collection of clinical samples and data including clinical information, tumor tissue and blood samples, we designed the Tongren-ophthalmology Research Association of Clinical Evaluation (TRACE), a database for the research of UM in China. The Surveillance, Epidemiology and End Results (SEER) Program of the National Cancer Institute is an important source of the longitudinal epidemiological study from 18 population-based registries, which covers nearly 28% of the population in America [9]. Our study compares the clinical features, treatment modalities and prognosis of UM in China and America via a comparative study of TRACE and SEER 18. The aim is to enhance understanding of the racial differences between UM patients and guide the design of effective prevention and treatment strategies.

## 2. Materials and Methods

### 2.1. The Study Subject

American population-based data were obtained from the SEER program (18 registries, diagnosed from 2000 to 2018, submitted on November 2020). Data retrieved from SEER included demographics, disease characteristics, the first course of treatment, and survival follow-up of different primary malignancies [12]. We identified UM patients according to the following inclusion criteria: (1) the International Classification of Diseases for Oncology 3 (ICD-O-3) site was limited to “eye and orbit”, (ICD-O-3 site code C690 through C699); (2) the histology was confirmed as “choroid melanoma” or “ciliary body melanoma” or “iris melanoma” (ICD-O-3 Histology code 872, 873, 874, 876, 877; and TNM 7/CS v0204+ code 067, 068, 077); (3) diagnosed since 2004 and later, when the AJCC 6th and AJCC 7th were available for tumor stages. The exclusion criteria were: (1) diagnosed at autopsy or death certificate, or lost to follow-up; (2) missing or unknown T stage, or coded as “Tx”; (3) missing or unknown M stage; (4) with synchronous metastasis, or diagnosed as M1 or Stage IV; (5) concomitant diagnosis of other malignancies. After application of inclusion and exclusion criteria, we obtained a cohort of 4066 American UM patients from the database. Since SEER is an open database providing anonymous information, no informed consent was needed.

Chinese UM patients were derived from the TRACE database. TRACE is a single-center database that includes Chinese UM patients referred from 32 provinces and managed at Beijing Tongren hospital from 2005 to 2020. The main diagnostic strategy is consistent with recommendations of the Collaborative Ocular Melanoma Study (COMS) [13]. The inclusion and exclusion criteria were consistent with those mentioned above. We assembled a sample of 1508 Chinese patients in TRACE. All patients have signed their informed consent, and the ethics committee of Beijing Tongren Hospital of Capital Medical University approved this study in accordance with the declaration of Helsinki.

### 2.2. Study Variables

Demographics, disease staging, treatment and outcome were extracted from both SEER and TRACE databases. Demographic data included age, sex, race and year of diagnosis. The patient’s age was categorized into “≤40”, “41–60”, “61–80” and “>80” years old. The race of patients was classified as “African”, “Asian”, “White” and “Other”. For comparison between two databases, patients were classified into “African American”, “Asian American”, “White American” in SEER and “Chinese” in TRACE. Clinical characteristics included the location of tumor, laterality, pathology, tumor diameter, tumor thickness and tumor stage. Tumors were staged according to AJCC 7th edition. BT and EN were the main treatments. We analyzed BT and EU, respectively, which were the most commonly applied modalities around the world. Other treatments such as laser, local resection and external beam radiotherapy were coded as “Other”. The outcome measure was overall survival (OS) which was calculated from the date of diagnosis to death. Patients were censored at the last follow-up. 

### 2.3. Study Design

**Figure 1:**
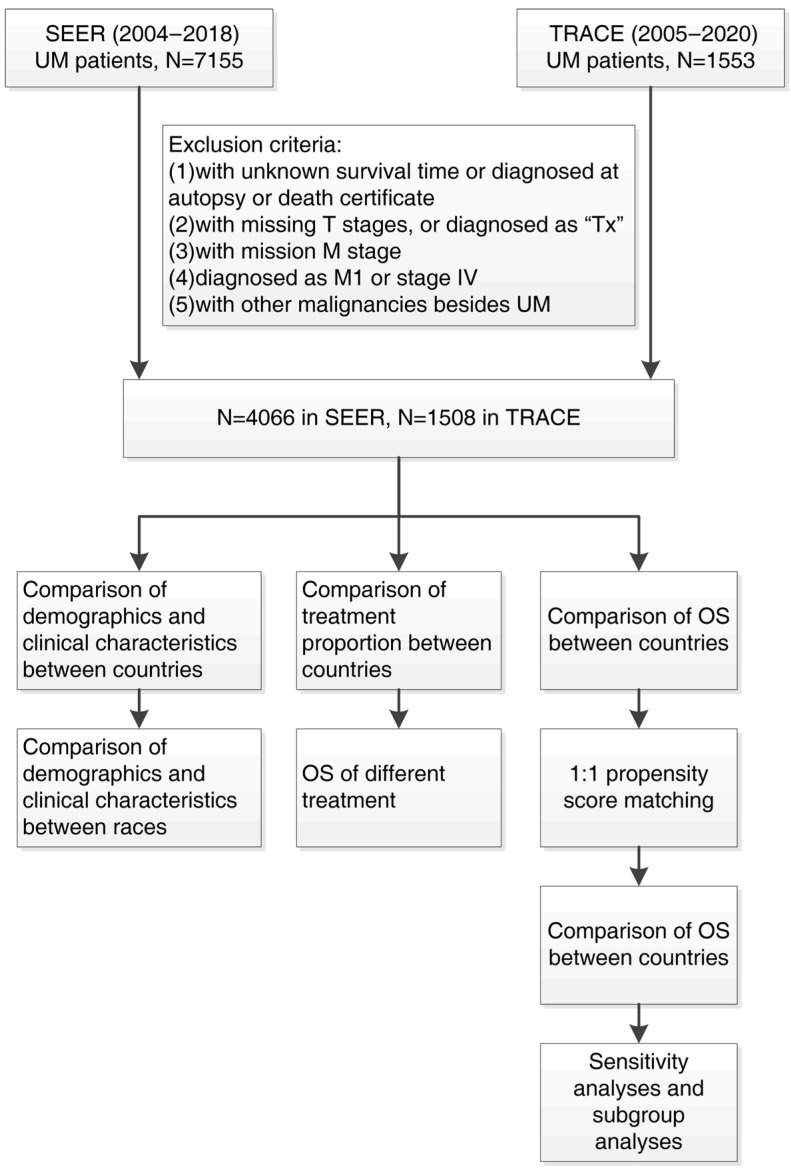
Flowchart of patients’ inclusion and analysis process.

As shown in Figure 1, demographic and clinical characteristics of UM were compared between different countries and racial groups. OS of patients receiving different treatments and diagnosed in different years were analyzed in two databases respectively. Proportion of patients receiving different treatments and their corresponding clinical characteristics were compared. In addition, trends of treatments over time were also studied. 

The OS of patients was compared between two databases. Survival-related variables were derived by univariable cox regression, then used for propensity score matching (PSM) and multivariable cox analysis. We performed 1:1 PSM between two databases to reduce the impact of potential confounding variables on prognosis. After PSM, the OS were compared between two databases using the overall dataset and then applying subgroup analysis. In order to determine variable responsible for differences in prognosis, we further performed sensitivity analyses, by matching survival-related variables between two databases separately and analyzing the OS. 

### 2.4. Statistical Analysis

Categorical variables were summarized by frequency, percentage and compared by Pearson’s chi-squared test and/or odd ratio (OR). Continuous variables were assessed by mean, standard deviation (SD) and compared by independent *t* tests. OS, 3-year survival and 5-year survival were analyzed using the Kaplan–Meier method, and the difference in prognosis was determined by log-rank test. Risks regression model was applied to identify predictors of prognosis. We incorporated factors with *p* value < 0.05 identified in univariable analyses to develop multivariable regression models. Multivariate regression was used to estimate the subhazard ratio and evaluate the association between variables and risk of prognosis. The univariable cox regression model was used to confirm independent prognostic factors and calculate hazard ratio (HR) and 95% confidence interval (CI). Statistical analyses were performed using R software (R Software Inc., San Francisco, CA, USA) version 4.1.0 (www.r-project.org, accessed on 18 May 2021) and SPSS software version 23.0 (IBM Corporation, Armonk, NY, USA). A two-side *p*-value < 0.05 was considered statistically significant.

## 3. Results

### 3.1. Population and Tumor Characteristics

Studied patients included 4066 patients from SEER and 1508 patients from TRACE. The demographic and clinicopathological characteristics were shown in Table 1. The mean age of Chinese patients in TRACE was 47.3 ± 12.5 years old (mean ± SD), younger than American patients in SEER, mean age 59.7 ± 14.8 years old (*p* < 0.001). Proportion of males and females were not significantly different between the two groups (*p* = 0.500). All patients had unilateral UM, with similar rates of left or right eye involvement (*p* = 0.583). Tumor pathology from TRACE and SEER included epithelioid cells (12.4% (108/869) vs. 22.5% (83/369)), mixed cells (38.9% (338/869) vs. 34.7% (128/369)), and spindle cells (48.7% (423/869) vs. 42.8% (158/369)), respectively (*p* < 0.001).

Choroid was the most common location of UM in both groups, while the proportion of ciliary body or iris in SEER was higher than that in TRACE (ciliary body: 9.03% vs. 4.18%; iris: 2.14% vs. 0.33%; *p* < 0.001). The stagings of SEER tumors were T stage 1 (1544, 38.0%), stage 2 (1541, 37.9%), stage 3 (712, 17.5%), stage 4 (269, 6.6%) or AJCC stage I (1436, 35.3%), stage II (1966, 48.4%) and stage III (664, 16.3%). In contrast, tumors in TRACE had more advanced staging with T stage 1 (212, 14.1%), stage 2 (614, 40.7%), stage 3 (557, 36.9%), stage 4 (125, 8.3%) or AJCC stage I (192, 12.7%), stage II (1076, 71.4%) and stage III (240, 15.9%). Tumors in TRACE had significantly larger basal diameter (12.0 ± 3.54 mm vs. 11.3 ± 8.27 mm, *p* < 0.001) and tumor thickness (7.1 ± 3.28 mm vs. 4.9 ± 3.01 mm, *p* < 0.001) than tumors in SEER.

### 3.2. Tumor Characteristics Disparity in Different Races

**Figure 2:**
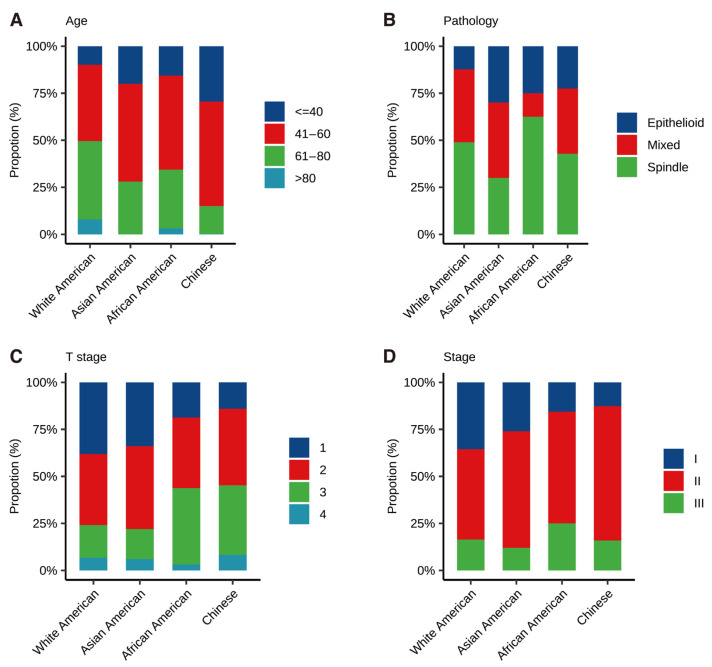
Distribution of different races in terms of age (**A**), pathology (**B**), T stage (**C**), and AJCC Stage (**D**).

We identified 3917 White Americans (96.3%), 32 African Americans (0.79%), 50 Asian Americans (1.23%) in SEER and 1508 Chinese (100%) in TRACE (Figure 2, Table 1).

Age at diagnosis was documented in Figure 2A and Tables S1–S4. Chinese was found to have the youngest age at diagnosis (47.3 ± 12.5 years old), followed by Asian Americans (51.9 ± 16.4 years old), African Americans (55.2 ± 15.8 years old) and White Americans (59.9 ± 14.7 years old) (*p* < 0.001). Comparison of pathology revealed that African Americans had significant higher proportion of spindle cell-type (62.5%) and lower proportion of mixed cell-type (12.5%), compared to Chinese (42.8% and 34.7%), Asian Americans (30.0% and 40.0%) and White Americans (48.9% and 38.8%)(Figure 2B, *p* < 0.001). As shown in Figure 2C,D, the proportion of tumors diagnosed at T Stage 1 was highest in White Americans, followed by Asian Americans, African Americans and Chinese (38.1%, 34.0%, 18.8% and 14.1%, respectively, *p* < 0.05). Besides, 75.8% and 78.0% of White Americans and Asian Americans were diagnosed at either T stage 1 or 2, compared to 54.8% and 56.3% of Chinese and African Americans, respectively. Of note, as documented in Table S3, when comparison was conducted among the same race, namely, Asian Americans and Chinese, only the T stage showed statistical difference (*p* < 0.001), while the age at diagnosis showed no significant difference (*p* = 0.05). In addition, when comparing three races within SEER, as depicted in Figure 2 and Tables S5–S8, only age at diagnosis exhibited statistical difference (*p* < 0.001). 

### 3.3. Trends of Treatment

BT was the most common modality in both countries (Table 2). Compared with SEER, patients from TRACE were more likely to undergo BT (73.2% vs. 50.9%, *p* < 0.001) and less likely to undergo EN (14.8% vs. 17.9%, *p* = 0.007). 

The clinical characteristics of patients in two databases receiving BT and EN were shown in Tables S9–S11, respectively. Among patients who underwent BT, tumors in TRACE showed a significant larger diameter and thickness than in SEER (diameter: TRACE = 11.9 ± 2.93 mm, SEER = 10.9 ± 8.36 mm, *p* < 0.001; thickness: TRACE = 6.82 ± 2.48 mm, SEER = 4.56 ± 2.81 mm, *p* < 0.001). Among patients who underwent EN, tumors in TRACE were significantly thicker than in SEER (10.8 ± 3.62 mm vs. 6.71 ± 3.30 mm, *p* < 0.001). 

**Figure 3:**
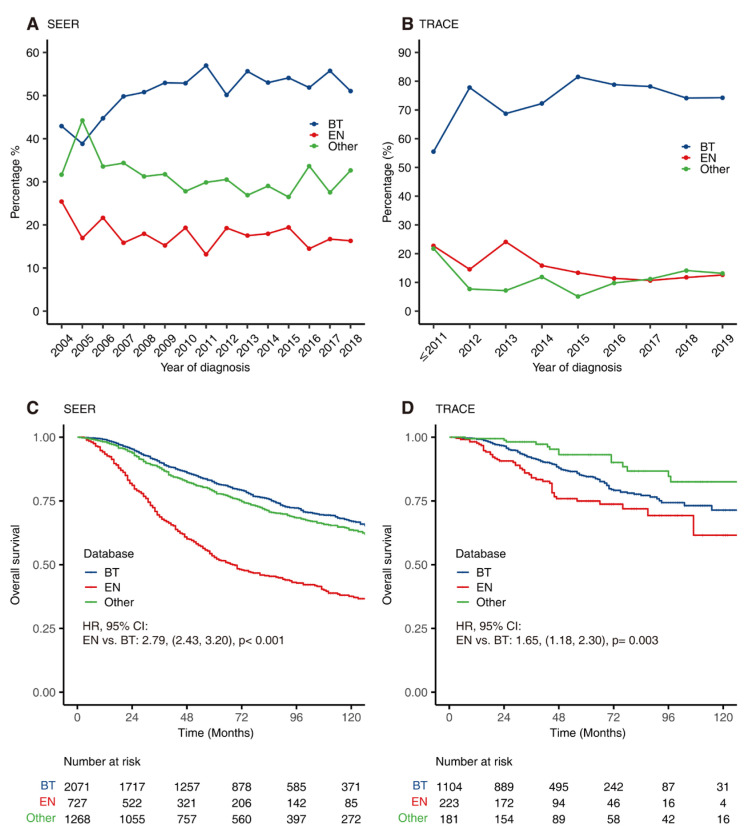
The trend of main treatments over time and their corresponding prognosis. The proportion of different treatments for patients with uveal melanomas over time in SEER (**A**) and TRACE (**B**). Kaplan–Meier curves for overall survival of patients receiving different treatments in SEER (**C**) and TRACE (**D**). BT = Brachytherapy, EN = Enucleation.

BT was carried out for 73% patients in TRACE. This trend was depicted in Figure 3A,B, showing more frequent use of BT over EN during the study period. The percentage of BT remained stable in SEER, while there was a shift away from EN toward BT in TRACE. In addition, as documented in Figure 3C,D, survival analysis of patients underwent different treatments and revealed that the BT group in both databases experienced a lower risk of death compared to the EN group.

### 3.4. Univariate and Multivariate Analysis of Prognosis-Related Factors

As shown in Table 3, variables were incorporated into univariate Cox regression analysis to screen for prognosis-related variables (those with *p* < 0.05) which were further included in multivariate cox regression analysis to identify independent prognostic factors. In SEER, univariate analysis revealed that older age, advanced T stage and AJCC stage, and pathology types of epithelioid and mixed cell-type were adverse prognostic factors, while in TRACE, the pathology type of epithelioid cell-type and advanced T stage and AJCC stage were found to be significant factors for worse prognosis. Multivariable Cox regression revealed that age and pathological type were independent prognostic factors of UM in both databases. 

### 3.5. Comparison of OS between SEER and TRACE

**Figure 4:**
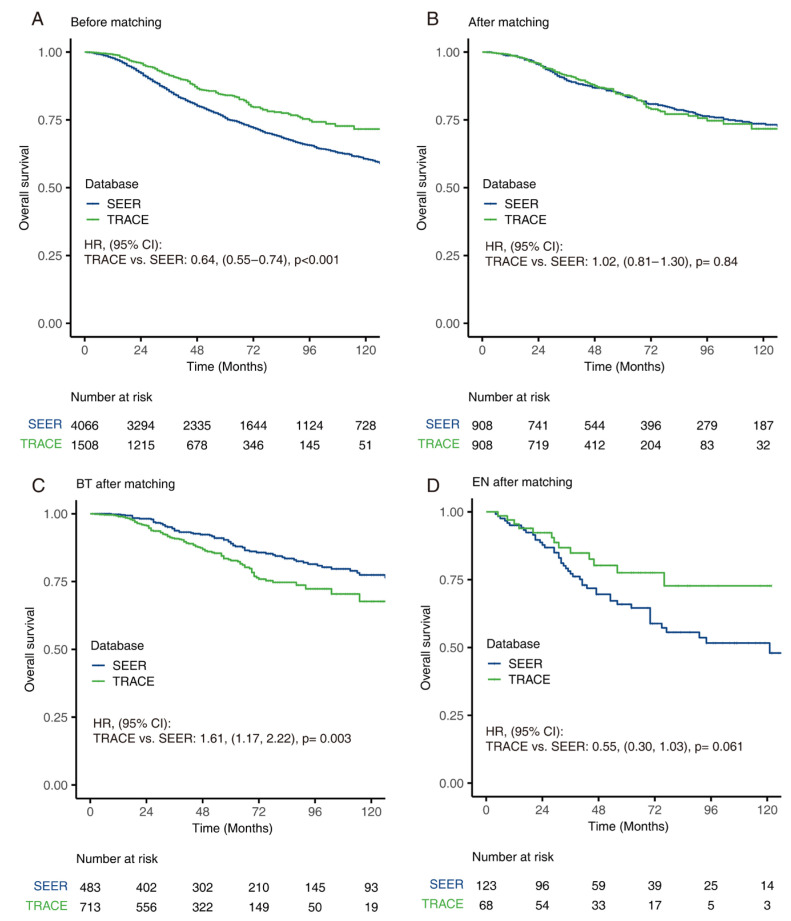
Survival comparison between SEER and TRACE. Kaplan–Meier curve of overall survival of patients with uveal melanomas grouped by database before matching (**A**) and after matching (**B**). Kaplan–Meier curves of overall survival for patients receiving brachytherapy (**C**) and enucleation (**D**), after matching between databases.

In general, patients in TRACE had better OS than those in SEER (Figure 4A, *p* < 0.001). Statistical adjustment by PSM was performed to eliminate the influence of confounding factors. Prognostic factors adjusted via PSM included age, sex, pathological type, T stage and AJCC Stage. After matching, there was no statistical difference in the distribution of these factors (Table 4). Analysis revealed that there was no longer a significant difference in both OS and disease-specific survival in TRACE and SEER after matching (Figure 4B and Supplementary Figure S1A). When comparing patients undergoing different treatments (Figure 4C,D and Figure S1B–E), the BT group and EN group showed significant differences between two databases after PSM. Patients underwent BT in SEER experienced better prognosis than in TRACE (*p* = 0.003) while patients who had EN had better prognosis in TRACE than SEER (*p* = 0.061). In addition, SEER exhibited a slight improvement in OS overtime (Figure S2A, *p* = 0.03); TRACE showed similar OS overtime (Figure S2B, *p* = 0.44).

**Figure 5:**
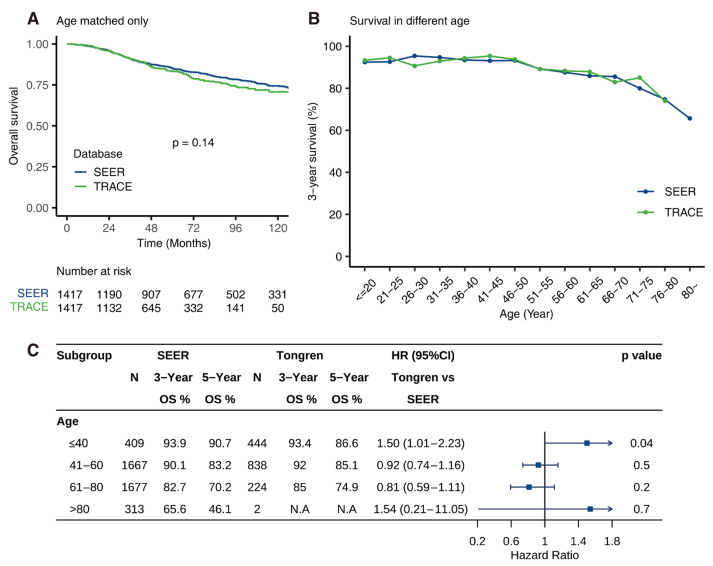
Age-related survival and age-stratified analysis. Comparison of 3-year survival and 5-year survival between SEER and TRACE for patients of different ages (**A**). The trend of 3-year survival with age (**B**). Kaplan–Meier curve of overall survival of patients after only matching age between two databases (**C**).

Sensitivity analysis was used to evaluate the factors that contributed to the prognostic difference. With the estimated propensity score using factors that were considered covariates in univariate analysis, including age, AJCC stage, T stage, pathology types, a one-to-one matched cohort was constituted (Figure 5A and Figure S3A–D). Two groups showed similar prognosis only when age was matched. Age-stratified subgroup analyses of two databases exhibited similar 3-year survival rates and 5-year survival rates, indicating that age was the main reason for better prognosis in TRACE. When matched for other factors, patients from TRACE still showed a better prognosis. 

### 3.6. Age-Dependent Survival Characteristic

As illustrated in Figure 5B,C, young patients (<40-year-old) in SEER experienced better 5-year survival than TRACE (90.7% vs. 86.6%, *p* = 0.04) over the study period and no apparent differences in 3-year survival were seen (93.9% vs. 93.4%, *p* = 0.04). In addition, older patients from TRACE (41–60 years old: 92%, 85.1%; 61–80 years old: 85%, 74.9%) experienced a better 3- and 5-year survival compared to SEER (41–60 years old: 90.1%, 83.2%; 61–80 years old: 82.7%, 70.2%). In addition, patients from both SEER and TRACE showed inverse correlation between age and 3-year and 5-year survival. 

## 4. Discussion

In this study, we compared the clinical characteristics, treatment modalities, and prognosis of UM patients between Chinese and American cohorts. We first observed that Chinese patients were diagnosed at a younger age than American patients (mean age 47 vs. 58 years), which was consistent with previous findings [6]. There was no significant difference in the age at diagnosis between Asian Americans and Chinese. This, at least in part, suggests that the age at diagnosis is associated with racial differences and less likely with environmental factors. There perhaps is an underlying genetic susceptibility or molecular mechanism that may influence the epidemiology of UM [9,14,15]. Future works would examine the bulk sequencing and single-cell sequencing to elucidate transcriptome and genome differences of Chinese patient with UM and those from the Cancer Genome Atlas. 

At diagnosis, Chinese patients had more advanced AJCC staging, significantly larger tumor base diameter and thickness than American patients including Asian Americans. This suggests a delay to diagnosis of UM in China [16,17,18,19]. We hypothesize improved education and awareness of UM among general and subspeciality ophthalmologists would enable earlier diagnosis of UM at routine eye screenings which in turn facilitates improved prognosis [20]. 

Moreover, we noted differences in the treatment of UM between China and America. BT is the most used modality in both countries, which is consistent with previous literature [21,22]. However, the proportion of patients receiving BT in TRACE were higher than SEER. In America, patients have access to a wider variety of treatments aside from BT, while BT is the primary eye-preserving modality available in China. Patients and physicians are careful to elect EN with primary UM in China.

In addition to treatment modality, the differences in treatment parameters between the two countries may affect the efficacy of radiotherapy. Echegaray et al. [23] reported when the mean radiation dose at the tumor apex varied between 62 and 104 Gy, the local recurrence rate decreased by 0.14% for each 1 Gy increase in dose. With tumor control as our primary objective, our center historically used an apical dose of 100 Gy, which is higher than the 85 Gy recommended by the COMS study [24]. However, higher radiation conferred more complication such as neovascular glaucoma and radiation retinopathy, which in turn prompted more secondary EN [25,26,27]. Recently, more emerging studies have found lower radiation doses can achieve satisfactory tumor control with less complications [28,29], which will likely shift our institutional practice in the future. 

The pattern of treatment selection of both countries demonstrates an increase in the use of BT as primary therapy. In terms of OS, there has been no significant improvement in China or America in the last two decades. Although there has been improvement in ocular preservation, with the recent approval of AU-011 in the European Union as the first novel virus-like drug conjugate therapy [30], survival of patients with metastatic disease is poor [1]. This is attributable to the fact that micro metastasis has likely already taken place at the time of diagnosis. Treatment of metastatic UM is a fundamental way to improve prognosis, which still requires further innovation, such as immunotherapy and targeted therapy [31]. One promising innovation is Tebentafusp, a novel class of T-cell receptor bispecific immunologic agents, demonstrated statistically and clinically meaningful OS benefits in Phase 3 clinical trials. Tebentafusp is currently approved by the U.S. Food and Drug Administration for the treatment of metastatic UM. 

Finally, we demonstrated patients in TRACE had better prognosis than SEER patients when matching for factors other than age. After matching for age, there was no longer a significant prognostic difference. Andreoli MT et al. showed a gradual increase of age at diagnosis over the years of the SEER database [32] and we noted the same trend for TRACE (Supplementary Figure S4). In Bishop K D’s [33] review of SEER data, he reported a 5-year survival of 78.4% in 7069 ocular melanoma patients. Our team [11] reported a 5-year survival of 84.0% in a Chinese cohort in analysis of 1500 individuals. This is corroborated by, Han Yue et al. [4]’s work, which revealed a 5-year all-cause mortality rate of 16% among Chinese patients. UM patients from TRACE were significantly younger than those at SEER. Xu Y et al. [34] previously demonstrated age as a predictor of patient survival for uveal melanoma based on SEER data, which is again demonstrated in our present study. We suspect younger age is associated with more robust immune system and therefore, is less likely to develop immune evasion of metastatic disease. Though the overall prognosis is better, younger patients have higher life expectancy and longer survival with tumor, and therefore, this results in a greater social burden of the disease [35]. Further studies of gene expression profile or genetic analysis between the two cohorts are warranted to evaluate underlying mechanism that account for early disease onset and difference in prognosis. 

## 5. Conclusions

We present the first study to compare the differences in clinical features, treatment modalities, providing supportive data in the comparative prognosis of UM patients in China and America. This work offers a world-wide insight into this rare malignancy. We found Chinese patients are younger at diagnosis, have more advanced AJCC at diagnosis and demonstrate higher survival compared to American patients. However, the survival advantage is not present after adjusting for age at diagnosis. Taken together, our work improves understanding of racial variation of UM to inform disease prevention, diagnosis, treatment and health policy development.

## Figures and Tables

**Table 1:** Demographic and clinical characteristics of patients with uveal melanoma from SEER and TRACE database.

	SEER (N = 4066)	TRACE (N = 1508)	*p* Value
Age:	59.7 (14.8)	47.3 (12.5)	<0.001
Sex:			0.500
Male	1944 (47.8%)	737 (48.9%)	
Female	2122 (52.2%)	771 (51.1%)	
Race:			0.000
African	32 (0.79%)	0 (0.00%)	
Asian	50 (1.23%)	1508 (100%)	
Other	67 (1.65%)	0 (0.00%)	
White	3917 (96.3%)	0 (0.00%)	
Location:			<0.001
Choroid	3612 (88.8%)	1440 (95.5%)	
Ciliary body	367 (9.03%)	63 (4.18%)	
Iris	87 (2.14%)	5 (0.33%)	
Laterality:			0.583
Left	2016 (49.7%)	736 (48.8%)	
Right	2042 (50.3%)	772 (51.2%)	
Pathology:			<0.001
Epithelioid	108 (12.4%)	83 (22.5%)	
Mixed	338 (38.9%)	128 (34.7%)	
Spindle	423 (48.7%)	158 (42.8%)	
T:			<0.001
1	1544 (38.0%)	212 (14.1%)	
2	1541 (37.9%)	614 (40.7%)	
3	712 (17.5%)	557 (36.9%)	
4	269 (6.6%)	125 (8.3%)	
Diameter	11.3 (8.27)	12.0 (3.54)	
Thickness	4.91 (3.01)	7.13 (3.28)	
M: 0	4066 (100%)	1508 (100%)	
Stage:			<0.001
I	1436 (35.3%)	192 (12.7%)	
II	1966 (48.4%)	1076 (71.4%)	
III	664 (16.3%)	240 (15.9%)	

**Table 2:** The number and proportion of patients receiving different main treatments in two databases.

	SEER (N = 4066)	TRACE (N = 1508)	OR	*p* Value
Brachytherapy:				<0.001
No	1995 (49.1%)	404 (26.8%)	Ref.	
Yes	2071 (50.9%)	1104 (73.2%)	2.63 [2.31; 3.00]	
Enucleation:				0.007
No	3339 (82.1%)	1285 (85.2%)	Ref.	
Yes	727 (17.9%)	223 (14.8%)	0.80 [0.68; 0.94]	
Other:				<0.001
No	2798 (68.8%)	1327 (88.0%)	Ref.	
Yes	1268 (31.2%)	181 (12.0%)	0.30 [0.25; 0.36]	

**Table 3:** Univariable and multivariable analyses about prognostic factors in SEER and TRACE databases.

	SEER				TRACE			
	Univariable	*p* Value	Multivariable	*p* Value	Univariable	*p* Value	Mulitvariable	*p* Value
Age								
≤40	Reference		Reference		Reference		Reference	
41–60	1.90 (1.39–2.59)	<0.001	1.88 (1.37–2.56)	<0.001	1.17 (0.85–1.62)	0.344	1.15 (0.83–1.60)	0.402
61–80	3.55 (2.62–4.81)	<0.001	3.53 (2.60–4.79)	<0.001	1.99 (1.34–2.98)	0.001	2.10 (1.40–3.14)	<0.001
>80	9.02 (6.52–12.48)	<0.001	8.62 (6.23–11.94)	<0.001	9.46 (1.3–68.77)	0.026	16.03 (2.18–118.16)	0.006
Sex								
Female	Reference				Reference			
Male	1.11 (0.99–1.25)	0.078			1.12 (0.85–1.47)	0.431		
Laterality								
Left	Reference				Reference			
Right	1.11 (0.99–1.24)	0.089			1.03 (0.78–1.35)	0.845		
Location								
Choroid	Reference		Reference		Reference		Reference	
Ciliary body	1.27 (1.06–1.51)	0.008	1.28 (1.06–1.53)	0.01	1.22 (0.64–2.3)	0.545	0.77 (0.37–1.59)	0.478
Iris	0.14 (0.04–0.42)	0.001	0.16 (0.05–0.50)	0.002	6.95 (0.96–50.15)	0.055	9.54 (1.28–71.15)	0.028
Pathology								
Spindle	Reference		Reference		Reference		Reference	
Epithelioid	3.1 (2.24–4.30)	<0.001	1.97 (1.41–2.73)	<0.001	2.6 (1.57–4.3)	<0.001	2.52 (1.51–4.20)	<0.001
Mixed	2.72 (2.11–3.50)	<0.001	1.84 (1.42–2.37)	<0.001	1.37 (0.8–2.33)	0.251	1.15 (0.67–1.98)	0.611
T								
T1	Reference		Reference		Reference		Reference	
T2	1.54 (1.33–1.79)	<0.001	1.19 (0.71–2.00)	0.511	1.58 (0.85–2.93)	0.149	0.65 (0.18–2.32)	0.509
T3	3.1 (2.63–3.66)	<0.001	2.14 (1.24–3.72)	0.007	3.35 (1.84–6.11)	<0.001	1.25 (0.35–4.40)	0.731
T4	4.56 (3.7–5.63)	<0.001	2.72 (1.50–4.93)	0.001	6.24 (3.22–12.1)	<0.001	1.51 (0.37–6.16)	0.564
Stage								
I	Reference		Reference		Reference		Reference	
II	1.64 (1.42–1.90)	<0.001	1.25 (0.74–2.11)	0.406	2.59 (1.32–5.01)	0.006	3.09 (0.76–12.65)	0.116
III	3.76 (3.19–4.42)	<0.001	1.42 (0.80–2.54)	0.234	6.36 (3.15–12.85)	<0.001	4.42 (0.95–20.67)	0.059

**Table 4:** Baseline characteristics of patients with uveal melanoma grouped by SEER and TRACE databases, before and after propensity score matching.

	Before Matching			After Matching		
	SEER	TRACE	*p* Value	SEER	TRACE	*p* Value
Age	59.7 (14.8)	47.3 (12.5)	<0.001	51.5 (11.8)	51.1 (11.4)	0.559
Sex:			0.500			0.963
Female	1944 (47.8%)	737 (48.9%)		439 (48.3%)	441 (48.6%)	
Male	2122 (52.2%)	771 (51.1%)		469 (51.7%)	467 (51.4%)	
Location:			<0.001			0.083
Choroid	3612 (88.8%)	1440 (95.5%)		890 (98.0%)	876 (96.5%)	
Ciliary body	367 (9.03%)	63 (4.18%)		17 (1.87%)	27 (2.97%)	
Iris	87 (2.14%)	5 (0.33%)		1 (0.11%)	5 (0.55%)	
Pathology:			<0.001			0.328
Epithelioid	108 (2.66%)	83 (5.50%)		14 (1.54%)	8 (0.88%)	
Mixed	338 (8.31%)	128 (8.49%)		36 (3.96%)	34 (3.74%)	
Spindle	423 (10.4%)	158 (10.5%)		58 (6.39%)	73 (8.04%)	
Unknown	3197 (78.6%)	1139 (75.5%)		800 (88.1%)	793 (87.3%)	
T:			<0.001			0.916
1	1544 (38.0%)	212 (14.1%)		194 (21.4%)	185 (20.4%)	
2	1541 (37.9%)	614 (40.7%)		457 (50.3%)	470 (51.8%)	
3	712 (17.5%)	557 (36.9%)		217 (23.9%)	216 (23.8%)	
4	269 (6.62%)	125 (8.29%)		40 (4.41%)	37 (4.07%)	
Stage:			<0.001			0.749
I	1436 (35.3%)	192 (12.7%)		190 (20.9%)	177 (19.5%)	
II	1966 (48.4%)	1076 (71.4%)		639 (70.4%)	650 (71.6%)	
III	664 (16.3%)	240 (15.9%)		79 (8.70%)	81 (8.92%)	
Stage:			<0.001			0.749

## Data Availability

The data can be shared up on request.

## Supplementary Materials

The following supporting information can be downloaded at: https://www.mdpi.com/article/10.3390/cancers14163960/s1, Figure S1: Kaplan–Meier curves of overall survival for patients receiving other treatments in two databases; Figure S2: Kaplan–Meier curves of overall survival for patients in two databases over the study period; Figure S3: Kaplan–Meier curve of overall survival of patients after only matching subtype, pathology, T stage and AJCC Stage between two databases; Figure S4: Age at diagnosis through the years of TRACE; Table S1: Comparison of clinical characteristics of tumors between different races; Table S2: Comparison of clinical characteristics of tumors between White Americans and Chinese; Table S3: Comparison of clinical characteristics of tumors between Asian Americans and Chinese; Table S4: Comparison of clinical characteristics of tumors between African Americans and Chinese; Table S5: Comparison of clinical characteristics of tumors between different races among SEER; Table S6: Comparison of clinical characteristics of tumors between White Americans and Asian Americans; Table S7: Comparison of clinical characteristics of tumors between White Americans and African Americans; Table S8: Comparison of clinical characteristics of tumors between Asian Americans and African Americans; Table S9: Comparison of clinical characteristics of tumors between SEER and TRACE in UM patients received BT; Table S10: Comparison of clinical characteristics of tumors between SEER and TRACE in UM patients received EN; Table S11: Comparison of clinical characteristics of tumors between SEER and TRACE in UM patients received other treatments except BT or EN.
