# Supplementary material for: Characteristics, Treatments, and Survival of Uveal Melanoma: A Comparison between Chinese and American Cohorts
Source: Cancers (Basel). 2022 Aug 17;14(16):3960. doi: 10.3390/cancers14163960 (PMC9406112; doi:10.3390/cancers14163960)
Supplement: Supplementary file 1 [file cancers-14-03960-s001.zip › cancers-1829775-supplementary.pdf]

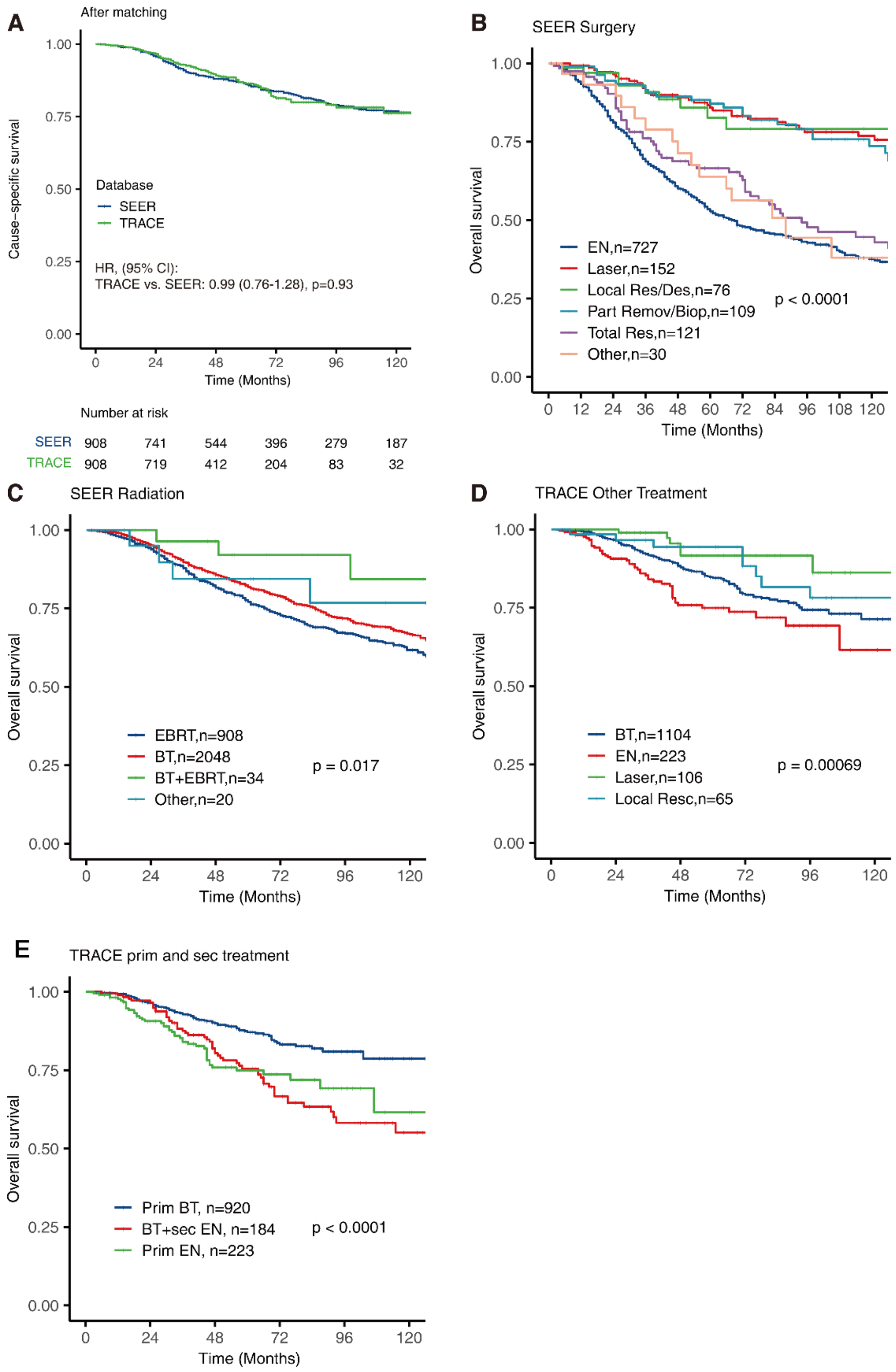

Supplementary Figure S1: Kaplan-Meier curves of overall survival for patients receiving other treatments in two databases. EN = Enucleation, Local Res = Local resection, Part Remov = Part removement, Total Res = Total resection,

BT = Brachytherapy, EBRT = External beam radiotherapy, Prim = Primary, Sec = Secondary.

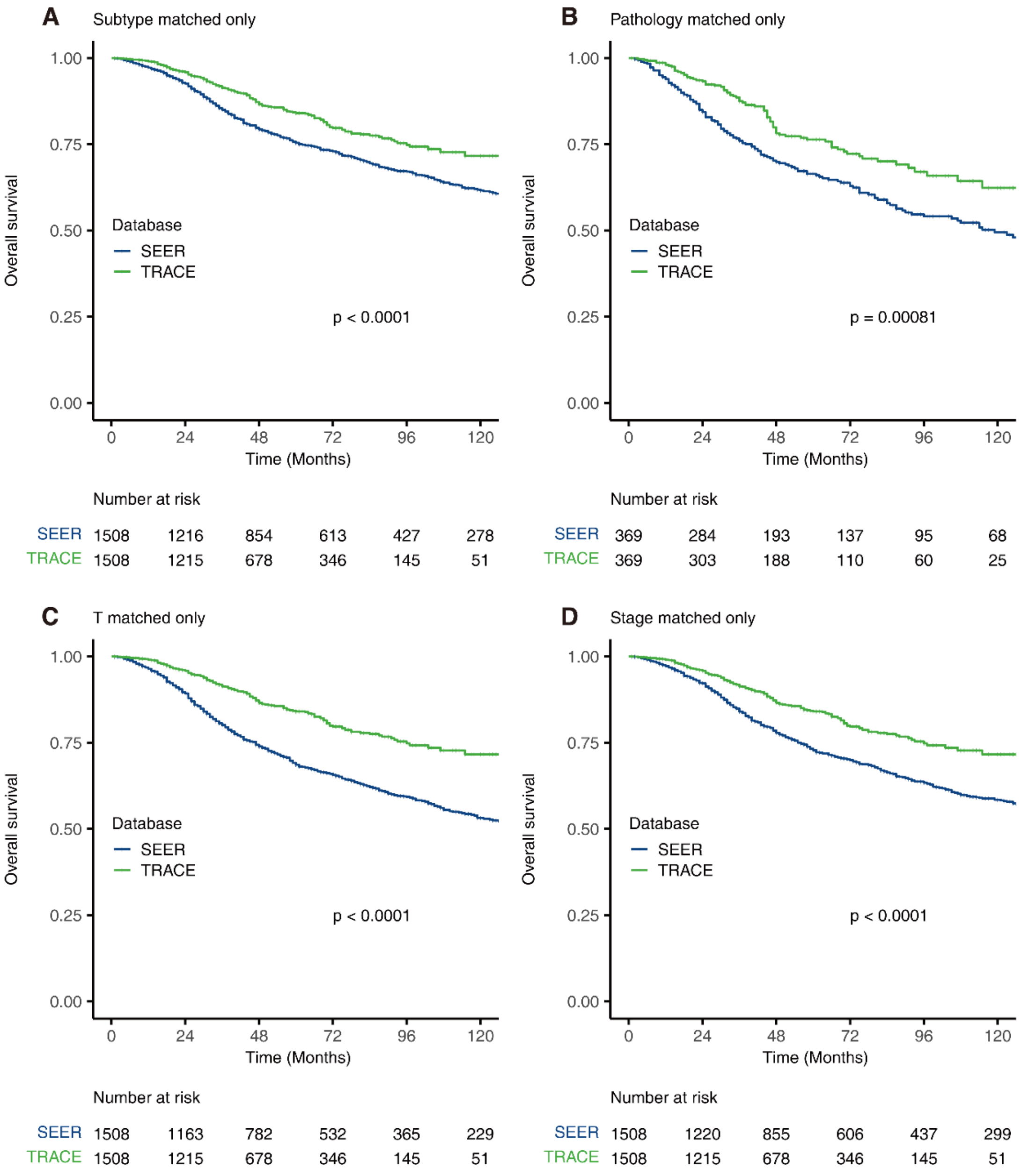

Supplementary Figure S2: Kaplan-Meier curves of overall survival for patients in two databases over the study period.

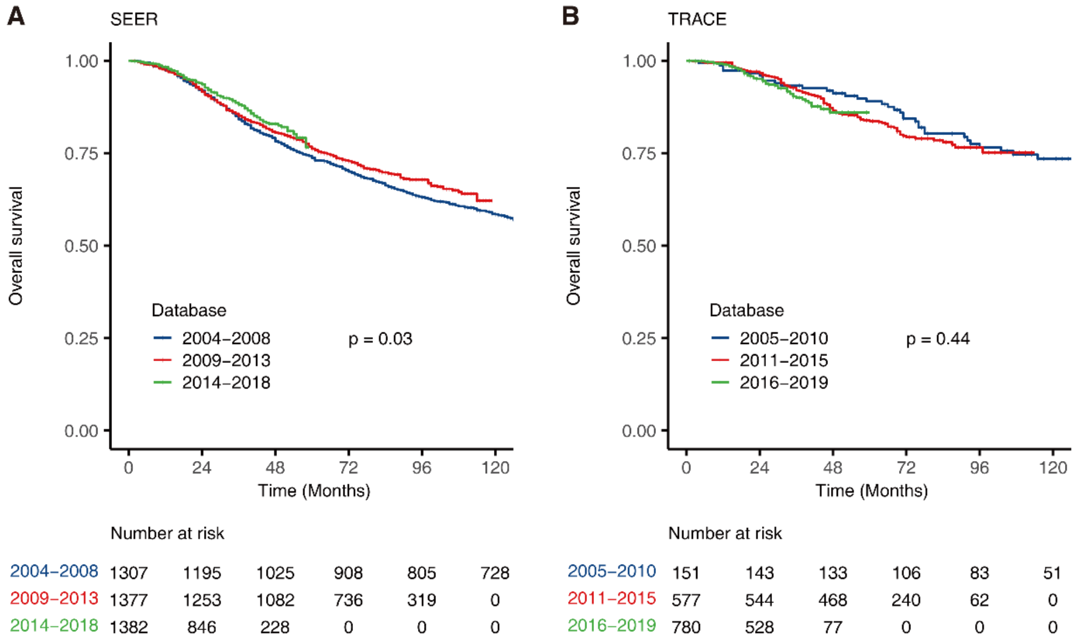

Supplementary Figure S3: Kaplan-Meier curve of overall survival of patients after only matching subtype, pathology, T stage, and AJCC Stage between two databases.

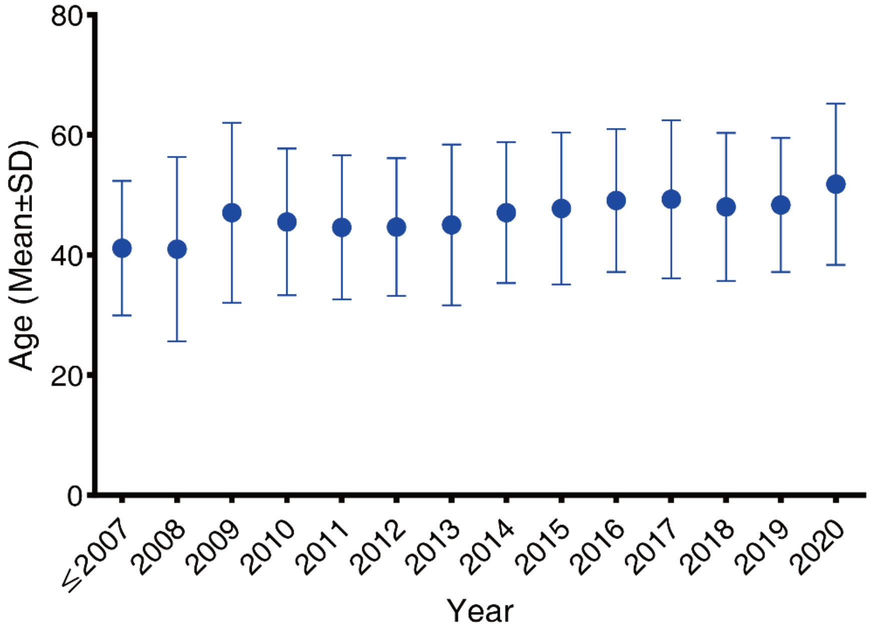

Supplementary Figure S4: Age at diagnosis through the years of TRACE.

Supplementary Table S1: Comparison of clinical characteristics of tumora between different races.

	White American	Asian American	African American	Chinese	*p* value
	N=3917	N=50	N=32	N=1508	
Age	59.9 (14.7)	51.9 (16.4)	55.2 (15.8)	47.3 (12.5)	<0.001
T:					<0.001
1	1494 (38.1%)	17 (34.0%)	6 (18.8%)	212 (14.1%)	
2	1478 (37.7%)	22 (44.0%)	12 (37.5%)	614 (40.7%)	
3	682 (17.4%)	8 (16.0%)	13 (40.6%)	557 (36.9%)	
4	263 (6.71%)	3 (6.00%)	1 (3.12%)	125 (8.29%)	
Diameter	11.3 (8.38)	11.1 (3.69)	11.0 (5.55)	12.0 (3.54)	0.018
Thickness	4.89 (3.00)	5.71 (3.01)	6.41 (3.27)	7.13 (3.28)	<0.001
Pathology:					<0.001
Epithelioid	103 (12.3%)	3 (30.0%)	2 (25.0%)	83 (22.5%)	
Mixed	325 (38.8%)	4 (40.0%)	1 (12.5%)	128 (34.7%)	
Spindle	409 (48.9%)	3 (30.0%)	5 (62.5%)	158 (42.8%)	
Stage:					<0.001
I	1392 (35.5%)	13 (26.0%)	5 (15.6%)	192 (12.7%)	
II	1881 (48.0%)	31 (62.0%)	19 (59.4%)	1076 (71.4%)	
III	644 (16.4%)	6 (12.0%)	8 (25.0%)	240 (15.9%)	

Supplementary Table S2: Comparison of clinical characteristics of tumors between White American and Chinese.

	White American	Chinese	*p* value
	N=3917	N=1508	
Age	59.9 (14.7)	47.3 (12.5)	<0.001
T:			<0.001
1	1494 (38.1%)	212 (14.1%)	
2	1478 (37.7%)	614 (40.7%)	
3	682 (17.4%)	557 (36.9%)	
4	263 (6.71%)	125 (8.29%)	
Diameter	11.3 (8.38)	12.0 (3.54)	<0.001
Thickness	4.89 (3.00)	7.13 (3.28)	<0.001
Pathology:			<0.001
Epithelioid	103 (12.3%)	83 (22.5%)	
Mixed	325 (38.8%)	128 (34.7%)	
Spindle	409 (48.9%)	158 (42.8%)	
Stage:			<0.001
I	1392 (35.5%)	192 (12.7%)	
II	1881 (48.0%)	1076 (71.4%)	
III	644 (16.4%)	240 (15.9%)	

Supplementary Table S3: Comparison of clinical characteristics of tumors between Asian American and Chinese.

	Asian American	Chinese	*p* value
	N=50	N=1508	
Age	51.9 (16.4)	47.3 (12.5)	0.050
T:			<0.001
1	17 (34.0%)	212 (14.1%)	
2	22 (44.0%)	614 (40.7%)	
3	8 (16.0%)	557 (36.9%)	
4	3 (6.00%)	125 (8.29%)	
Diameter	11.1 (3.69)	12.0 (3.54)	0.219
Thickness	5.71 (3.01)	7.13 (3.28)	0.023
Pathology:			0.715
Epithelioid	3 (30.0%)	83 (22.5%)	
Mixed	4 (40.0%)	128 (34.7%)	
Spindle	3 (30.0%)	158 (42.8%)	
Stage:			0.023
I	13 (26.0%)	192 (12.7%)	
II	31 (62.0%)	1076 (71.4%)	
III	6 (12.0%)	240 (15.9%)	

Supplementary Table S4: Comparison of clinical characteristics of tumors between African American and Chinese.

	African American	Chinese	*p* value
	N=32	N=1508	
Age	55.2 (15.8)	47.3 (12.5)	0.008
T:			0.671
1	6 (18.8%)	212 (14.1%)	
2	12 (37.5%)	614 (40.7%)	
3	13 (40.6%)	557 (36.9%)	
4	1 (3.12%)	125 (8.29%)	
Diameter	11.0 (5.55)	12.0 (3.54)	0.410
Thickness	6.41 (3.27)	7.13 (3.28)	0.321
Pathology:			0.424
Epithelioid	2 (25.0%)	83 (22.5%)	
Mixed	1 (12.5%)	128 (34.7%)	
Spindle	5 (62.5%)	158 (42.8%)	
Stage:			0.250
I	5 (15.6%)	192 (12.7%)	
II	19 (59.4%)	1076 (71.4%)	
III	8 (25.0%)	240 (15.9%)	

Supplementary Table S5: Comparison of clinical characteristics of tumors between different races among SEER.

	White American	Asian American	African American	*p* value
	N=3917	N=50	N=32	
Age	59.9 (14.7)	51.9 (16.4)	55.2 (15.8)	<0.001
T:				0.050
1	1494 (38.1%)	17 (34.0%)	6 (18.8%)	
2	1478 (37.7%)	22 (44.0%)	12 (37.5%)	
3	682 (17.4%)	8 (16.0%)	13 (40.6%)	
4	263 (6.71%)	3 (6.00%)	1 (3.12%)	
Diameter	11.3 (8.38)	11.1 (3.69)	11.0 (5.55)	0.980
Thickness	4.89 (3.00)	5.71 (3.01)	6.41 (3.27)	0.024
Pathology:				0.140
Epithelioid	103 (12.3%)	3 (30.0%)	2 (25.0%)	
Mixed	325 (38.8%)	4 (40.0%)	1 (12.5%)	
Spindle	409 (48.9%)	3 (30.0%)	5 (62.5%)	
Stage:				0.047
I	1392 (35.5%)	13 (26.0%)	5 (15.6%)	
II	1881 (48.0%)	31 (62.0%)	19 (59.4%)	
III	644 (16.4%)	6 (12.0%)	8 (25.0%)	

Supplementary Table S6: Comparison of clinical characteristics of tumors between White American and Asian American.

	White American	Asian American	*p* value
	N=3917	N=50	
Age	59.9 (14.7)	51.9 (16.4)	0.001
T:			0.862
1	1494 (38.1%)	17 (34.0%)	
2	1478 (37.7%)	22 (44.0%)	
3	682 (17.4%)	8 (16.0%)	
4	263 (6.71%)	3 (6.00%)	
Diameter	11.3 (8.38)	11.1 (3.69)	0.801
Thickness	4.89 (3.00)	5.71 (3.01)	0.169
Pathology:			0.212
Epithelioid	103 (12.3%)	3 (30.0%)	
Mixed	325 (38.8%)	4 (40.0%)	
Spindle	409 (48.9%)	3 (30.0%)	
Stage:			0.145
I	1392 (35.5%)	13 (26.0%)	
II	1881 (48.0%)	31 (62.0%)	
III	644 (16.4%)	6 (12.0%)	

Supplementary Table S7: Comparison of clinical characteristics of tumors between White American and African American.

	White American N=3917	African American N=32	*p* value
Age	59.9 (14.7)	55.2 (15.8)	0.102
T:			0.008
1	1494 (38.1%)	6 (18.8%)	
2	1478 (37.7%)	12 (37.5%)	
3	682 (17.4%)	13 (40.6%)	
4	263 (6.71%)	1 (3.12%)	
Diameter	11.3 (8.38)	11.0 (5.55)	0.804
Thickness	4.89 (3.00)	6.41 (3.27)	0.041
Pathology:			0.182
Epithelioid	103 (12.3%)	2 (25.0%)	
Mixed	325 (38.8%)	1 (12.5%)	
Spindle	409 (48.9%)	5 (62.5%)	
Stage:			0.055
I	1392 (35.5%)	5 (15.6%)	
II	1881 (48.0%)	19 (59.4%)	
III	644 (16.4%)	8 (25.0%)	

Supplementary Table S8: Comparison of clinical characteristics of tumors between Asian American and African American.

	Asian American	African American	*p* value
	N=50	N=32	
Age	51.9 (16.4)	55.2 (15.8)	0.378
T:			0.083
1	17 (34.0%)	6 (18.8%)	
2	22 (44.0%)	12 (37.5%)	
3	8 (16.0%)	13 (40.6%)	
4	3 (6.00%)	1 (3.12%)	
Diameter	11.1 (3.69)	11.0 (5.55)	0.932
Thickness	5.71 (3.01)	6.41 (3.27)	0.445
Pathology:			0.424
Epithelioid	3 (30.0%)	2 (25.0%)	
Mixed	4 (40.0%)	1 (12.5%)	
Spindle	3 (30.0%)	5 (62.5%)	
Stage:			0.233
I	13 (26.0%)	5 (15.6%)	
II	31 (62.0%)	19 (59.4%)	
III	6 (12.0%)	8 (25.0%)	

Supplementary Table S9: Comparison of clinical characteristics of tumors between SEER and TRACE in UM patients received BT.

	SEER	TRACE	*p* value
	N=2071	N=1104	
Age	59.4 (14.7)	47.3 (12.2)	<0.001
Sex:			0.814
Female	1010 (48.8%)	544 (49.3%)	
Male	1061 (51.2%)	560 (50.7%)	
T:			<0.001
1	859 (41.5%)	104 (9.42%)	
2	855 (41.3%)	535 (48.5%)	
3	280 (13.5%)	429 (38.9%)	
4	77 (3.72%)	36 (3.26%)	
Diameter	10.9 (8.36)	11.9 (2.93)	<0.001
Thickness	4.56 (2.81)	6.82 (2.48)	<0.001
Pathology:			<0.001
Epithelioid	9 (8.33%)	33 (34.7%)	
Mixed	30 (27.8%)	34 (35.8%)	
Spindle	69 (63.9%)	28 (29.5%)	
Stage:			<0.001
I	798 (38.5%)	96 (8.70%)	
II	1075 (51.9%)	911 (82.5%)	
III	198 (9.56%)	97 (8.79%)	

Supplementary Table S10: Comparison of clinical characteristics of tumors between SEER and TRACE in UM patients received EN.

	SEER	TRACE	*p* value
	N=727	N=223	
Age	59.8 (15.2)	47.0 (12.9)	<0.001
Sex:			0.541
Female	320 (44.0%)	104 (46.6%)	
Male	407 (56.0%)	119 (53.4%)	
T:			<0.001
1	152 (20.9%)	5 (2.24%)	
2	199 (27.4%)	36 (16.1%)	
3	237 (32.6%)	98 (43.9%)	
4	139 (19.1%)	84 (37.7%)	
Diameter	13.8 (9.36)	14.8 (3.99)	0.094
Thickness	6.71 (3.30)	10.8 (3.62)	<0.001
Pathology:			0.038
Epithelioid	69 (15.2%)	38 (17.6%)	
Mixed	213 (47.0%)	79 (36.6%)	
Spindle	171 (37.7%)	99 (45.8%)	
Stage:			<0.001
I	137 (18.8%)	4 (1.79%)	
II	288 (39.6%)	98 (43.9%)	
III	302 (41.5%)	121 (54.3%)	

Supplementary Table S11: Comparison of clinical characteristics of tumors between SEER and TRACE in UM patients received other treatments except BT or EN.

	SEER	TRACE	*p* value
	N=1268	N=181	
Age	60.1 (14.6)	47.1 (13.9)	<0.001
Sex:			0.913
Female	614 (48.4%)	89 (49.2%)	
Male	654 (51.6%)	92 (50.8%)	
T:			<0.001
1	533 (42.0%)	103 (56.9%)	
2	487 (38.4%)	43 (23.8%)	
3	195 (15.4%)	30 (16.6%)	
4	53 (4.18%)	5 (2.76%)	
Diameter	10.5 (7.16)	8.90 (3.66)	<0.001
Thickness	4.58 (2.84)	4.44 (3.34)	0.601
Pathology:			0.055
Epithelioid	30 (9.74%)	12 (20.7%)	
Mixed	95 (30.8%)	15 (25.9%)	
Spindle	183 (59.4%)	31 (53.4%)	
Stage:			0.012
I	501 (39.5%)	92 (50.8%)	
II	603 (47.6%)	67 (37.0%)	
III	164 (12.9%)	22 (12.2%)	
